# Green tea polyphenol treatment attenuates atherosclerosis in high-fat diet-fed apolipoprotein E-knockout mice via alleviating dyslipidemia and up-regulating autophagy

**DOI:** 10.1371/journal.pone.0181666

**Published:** 2017-08-04

**Authors:** Shibin Ding, Jinjin Jiang, Pengxin Yu, Guofu Zhang, Guanghui Zhang, Xiaoting Liu

**Affiliations:** 1 Department of Nutrition and Food Hygiene, School of Public Health, Xinxiang Medical University, Xinxiang, Henan Province, PR, China; 2 Henan Collaborative Innovation Center of Molecular Diagnosis and Laboratory Medicine, Xinxiang Medical University, Xinxiang, Henan Province, PR, China; 3 School of Public Health, Capital Medical University, Beijing, PR, China; Max Delbruck Centrum fur Molekulare Medizin Berlin Buch, GERMANY

## Abstract

Background: Green tea polyphenol (GTP) is a polyphenol source from green tea that has drawn wide attention owing to epidemiological evidence of its beneficial effects in the prevention of cardiovascular disease; the underlying molecular mechanisms of these effects are not well understood. This study aimed to investigate the effects of GTP treatment on autophagy regulation in the vessel wall and lipid metabolism of HFD-fed male ApoE-knockout mice. Methods: Adult male ApoE-knockout mice (n = 30) fed with a high-fat diet (HFD) were treated with either vehicle or GTP (3.2 or 6.4 g/L) administered via drinking water for 15 weeks, and C57BL/6J mice fed with standard chow diet (STD) were used as the control group. Metabolic parameters, expression of key mRNAs and proteins of hepatic lipid metabolism and autophagy in the vessel wall of mice were determined after the 15-week treatment. Results: A HFD induced atherosclerosis formation and lipid metabolism disorders as well as reduced autophagy expression in the vessel wall of ApoE-knockout mice, but GTP treatment alleviated the lipid metabolism disorders, decreased the oxLDL levels in serum, and increased the mRNA and protein expressions of hepatic PPARα and autophagy markers (LC3, Beclin1 and p62) in the vessel wall of ApoE-knockout mice. Conclusions: Our findings suggest that GTP supplementation showed marked suppression of atherogenesis through improved lipid metabolism as well as through a direct impact on oxLDL and autophagy flux in the vessel wall.

## Introduction

The prevalence of atherosclerosis has dramatically increased worldwide in the past several decades. Atherosclerosis is a multi-factorial disease characterized by excessive cholesterol deposition, proliferation of the smooth muscle cells, intimal thickening resulting in thrombus formation and stroke [1]. Atherosclerotic plaque destabilization and rupture can lead to serious damage and even death from acute coronary syndromes, and atherosclerosis is now recognized as a serious public health problem [2]. The endothelium, formed by endothelial cells located at the interior surface to the blood vascular wall, is an effective protective barrier between circulating blood and tissues. However, the abnormal accumulation of lipids leads to endothelial dysfunction and endothelial cell death that precedes atherogenesis [3].

Autophagy is an evolutionarily conserved cellular catabolic process responsible for maintaining cellular homeostasis that plays pivotal roles in metabolism, cell death, and differentiation through a lysosome-dependent pathway [4]. Accumulated evidence shows that the autophagy process is important in atherosclerosis; autophagy modulation plays an important role in vascular disease and has potential as a new therapeutic approach to treat atherosclerosis [5, 6]. However, the precise role of autophagy regulation and its relationship with atherosclerosis need to be further clarified. Green tea polyphenol (GTP), a type of flavonoid, is usually consumed via drinking a green tea beverage by people in many Asian countries and has a variety of beneficial roles, such as anti-oxidant function and regulation of lipid metabolism. Green tea intake has also been shown to be useful for prevention of cardiovascular disease [7, 8]; an increase in consumption of green tea (1 cup/day) was associated with a 10% decrease in the risk of developing coronary artery disease [9], but the mechanism is still not clear. Previous studies demonstrated that EGCG (epigallocatechin-3-gallate), a major ingredient of green tea polyphenol, could stimulate autophagy in vascular endothelial cell lines and human endothelial cells [10, 11]. Moreover, EGCG markedly reduced the formation of atherosclerotic lesions in animal models of atherosclerosis [12, 13]. These studies indicate that GTP and EGCG may have the potential to protect against atherosclerosis via regulating autophagy in vascular endothelial cells.

Accumulating evidence demonstrates that dyslipidemia, including high concentrations of cholesterol and low concentrations of HDL in plasma, is a major risk factor for coronary artery disease and signficantly contributes to arterial plaque formation. Green tea and tea constituents have a potential protective effect against coronary artery disease that may be due to lowering lipid levels [14, 15]. Animal studies also support the finding that GTP could improve lipid metabolism disorders [16, 17]. Our previous study has shown that GTP has beneficial health effects such as reducing fat deposits in high-fat fed rats [18]. All these findings support the hypothesis that green tea and tea constituents may prevent atherosclerosis via lipid lowering in humans. Peroxisome proliferator-activated receptors (PPARs) are nuclear hormone receptors and include three PPAR subtypes (alpha, delta/beta, and gamma) that function as transcription factors [19]. PPARα is expressed mainly in the liver and muscle, and the regulation of PPARα by EGCG has been suggested as a potential mechanism of its hypolipidemic and antiobesogenic effects [20]. Thus, exploring the effect of GTP treatment on the critical regulator of hepatic lipid metabolism in ApoE-knockout mice could further explain the positive effects of GTP against atherosclerosis from the standpoint of lipid homeostasis.

In this study, we investigated the mechanism of GTP-induced autophagy and its role in accumulation of lipids in vascular endothelial cells, as well as in dyslipidemia in ApoE-knockout mice. In this study we first demonstrate that GTP could alleviate atherosclerosis via stimulating autophagy in high-fat diet (HFD)-fed ApoE-knockout mice. In addition, our study addresses the role of hepatic PPARα in the process of hepatic lipid reduction and alleviation of atherosclerosis after GTP treatment.

## Materials and methods

### Chemicals and reagents

GTP (purity>98%, including catechin >90% and EGCG >70%) was purchased from Fuzhou Rimian Inc. (Fuzhou, FuJian, China). Assay kits for the analysis of total cholesterol (TC), triglycerides (TG), high-density lipoprotein cholesterol (HDL), low-density lipoprotein cholesterol (LDL) and Oxidized LDL (oxLDL) in serum were purchased from BIOSINO Biotechnology and Science Inc. (Beijing, China). TriZOL and the oligonucleotides for PCR were obtained from Invitrogen Inc. (Carlsbad, CA, USA), and the real-time quantitative PCR kit was purchased from TAKARA Bio Inc. (Otsu, Shiga, Japan). Anti-β-actin was obtained from Abcam (Cambridge, UK); anti-LC3B and anti-p62 antibodies were purchased from Cell Signaling Technology (Billerica, MA, USA). Anti-PPARα and secondary antibodies were obtained from Abcam (Cambridge, UK).

### Experimental procedures

Animal experimental protocols used for the study have been approved by the Animal Care and Ethical Use Committee of Xinxiang Medical University, in accordance to the guidelines for care and use of animals established by Xinxiang Medical University.

Thirty 8-week-old male ApoE^-/-^ (ApoE-knockout, 18–24 g) mice and ten C57BL/6J mice (17–23 g) were obtained from Vital River Laboratory Animal Technology Co. Ltd (Beijing, China). Mice were provided with a standard rodent chow diet and distilled water *ad libitum* and housed at 21±3°C, 60±10%-relative humidity, and exposed to 12 h light-12 h dark cycles.

After one week’s acclimation, ApoE^-/-^ mice were divided randomly into three groups (n = 10 for each group), and treated for 15 weeks as follows: (1) ApoE^-/-^/Control group was fed a high-fat diet (HFD); (2) ApoE^-/-^/GTP-L group was fed a HFD and administered GTP (3.2 g/L); (3) ApoE^-/-^/ GTP-H group received a high-fat diet (HFD) and was administered GTP (6.4 g/L). Ten C57BL/6J mice were fed a rodent standard chow diet (STD) as the C57BL/6J/Control group. The energy supply of the two diets was described in our previous study [21]; the ingredient composition of HFD fed to mice was as follows (w/w): standard chow, 60%; custard powder, 8%; lard, 12%; sugar, 12%; peanut power, 6%; and milk 1%. Mice were administered with either vehicle or GTP (3.2 or 6.4 g/L) via drinking water for 15 consecutive weeks. The dose of GTP (3.2 g/L) in drinking water reduced fat deposits in HFD-fed Wistar rats in our previous study[18].

At the end of the experiment, all mice were euthanized after being food -deprived for 12 h. Serum was prepared by centrifugation at 1000 × g for 10 min (Eppendorf 5810R, Hamburg, Germany). The liver tissues and aortas were isolated, the fat and adventitia were thoroughly cleaned under a dissecting microscope, and the remaining tissue was immediately frozen in liquid nitrogen and stored at -80°C until analysis. The white adipose tissue (WAT) coefficient was also calculated (subcutaneous fat pad weight × 100/body weight).

### Serum and hepatic lipid parameters

At the end of the treatment period (15 weeks), blood was collected from 12-h-fasted mice and was used to obtain serum by centrifugation. Serum concentrations of TG, TC, HDL, LDL and oxLDL were assayed using enzymatic colorimetric assays (BIOSINO Biotechnology and Science Inc., Beijing, China). The lipids were extracted from liver tissues as previously described [22], and then cholesterol and triglyceride levels were determined using serum cholesterol and triglyceride determination kits.

### Atherosclerotic lesion analysis

For atherosclerotic lesion area analysis, aortic root sections were stained with hematoxylin and eosin, and the total lesion areas were quantified as previously described [23]. The aortic roots were cut and fixed in formaldehyde, embedded in paraffin, and 5-μm sections were prepared and stained with hematoxylin and eosin. Aortic sinuses were embedded in OCT embedding medium, and cryosections (5 μm) were stained with Oil-red O. Images of sections were obtained by a digital camera (Olympus BX40, Hamburg, Germany) with an HMIAS-2000 medical imaging system and analyzed using Image Pro Plus 6.0. The lesion area of each mouse is described as the percentage of total luminal surface.

### Hematoxylin and eosin (HE) staining of liver tissue in mice

Aortic vessels and liver tissue samples from mice of the four groups were fixed in 4% paraformaldehyde and embedded in paraffin, sliced at 5-μm thickness, and stained with HE.

### Real-time PCR

The mRNA expression levels of *LC3*, *p62/SQSTM1*, *Beclin-1* and *TFEB* in aorta tissues and *PPARα*, *PPARγ*, *SREBP1C* and *FAS* in liver tissues were detected by quantitative real-time PCR. The primer sequences are given in Table 1. Brifly, total RNA from aorta and liver tissues was extracted by Trizol, and 1 μg of RNA was used to generate cDNA using a Primerscript RT Reagent Kit (Takara Bio). Reverse transcription reactions (RT) were performed according to the manufacturer’s instructions. The qRT-PCR analysis was performed in triplicate for target mRNAs using a qPCR SYBR Green Mix kit (Takara Bio), and the PCR conditions were as follows: 1 cycle of 95°C, 5 s; 40 cycles of 95°C, 10 s; 57°C, 30 s. The fluorescent signals of SYBR Green were subjected to cDNA analysis using an ABI 7900HT machine (Applied Biosystems, Forster, CA, USA). Melting curve analysis was performed to confirm specificity. The 2^-ΔΔCT^ method was used for the semi-quantitative PCR analysis. Target RNA levels were normalized to β-actin mRNA.

**Table 1:** Primer sequences used for real-time PCR.

Gene	Forward primer (5`-3`)	Reverse primer (5`-3`)
*TFEB*	ATCAACACCCCTGTCCACTT	GAACCTTCTGATGCTGGGACT
*LC3*	CGAGCTCATCAAGATAATCAGAC	TTCCTCCTG GTGAATGGGC
*p62/SQSTM1*	AGGATGGGGACTTGGTTGC	TCAC AGATCACATTGGGGTGC
*Beclin-1*	CTGAAACTGGACACGAGCTTCAAG	TGTGGTAAGTAATGGAGCTGTGAGT
*PPARα*	GGAGTGCAGCCTCAGCCAAGTT	AGGCCACAGAGCGCTAAGCTGT
*PPARγ*	ACGCGGGCTGAGAAGTCACG	AGTTGGTGGGCCAGAATGGCA
*SREBP-1C*	GGCACTAAGTGCCCTCAACCT	TGCGCAGGAGATGCTATCTCCA
*FAS*	CCTGGATAGCATTCCGAACCT	AGCACATCTCGAAGGCTACACA
*β-actin*	TTCGTTGCCGGTCCACACCC	GCTTTGCACATGCCGGAGCC

TFEB, the transcription factor EB; LC3, microtubule-associated protein 1A/1B-light chain 3; PPARα, peroxisome proliferator-activated receptor-alpha; PPARγ, peroxisome proliferator-activated receptor γ; SREBP-1C, the sterol regulatory element binding protein-1C; FAS, fatty acid synthase

### Protein extraction and Western blot analysis

In brief, aortal vessels and liver tissues were homogenized in ice-cold Tris buffer (0.01 M Tris, pH 7.4) and then lysed in extraction buffer containing 50 mmol/l Tris–HCl (pH 8.0), 150 mmol/l NaCl, 1% Nonidet-P40, 1% sodium deoxycholate, 0.1% SDS, 0.1 mmol/l dithiothreitol, 0.05 mmol/l phenylmethylsulphonyl fluoride, 0.002 mg/ml aprotinin, 0.002 mg/ml leupeptin, and 1 mmol/l NaVO_3_ [24]. Proteins of aortal vessels and liver tissues were separated by electrophoresis in a 10% or 12% SDS-polyacrylamide gel, and then were transferred onto a PVDF membrane (0.22 μm/0.45 μm, Bio-Rad). Blots were first incubated with a monoclonal antibody against β-actin (1:10000, Sigma-Aldrich Chemical Company), anti-LC3B (1:1000, Cell Signaling Technology), p62/SQSTM1 (1:1000, Cell Signaling Technology) and PPARα (1:1000, Abcam Limited, Cambridge) at 4°C for 12 h. After primary antibody incubation, the membranes were washed three times in washing buffer for 10 min each and then incubated with the respective horseradish peroxidase-conjugated secondary antibodies (1:5000; Beyotime, Haimen, Jiangsu, China) for 1 h at room temperature. The densities of blots were determined using an ECL detection system (Syngen, Cambridge, UK) and results were analyzed by Chemidoc-Quantity-One software (Bio-Rad Laboratories).

### Statistical analysis

Values are presented as the means ± SD and were analyzed using Analysis of Variance (ANOVA) followed by multiple comparisons using Bonferroni or Dunnett’s test as appropriate. *P* values less than 0.05 were considered significant. All statistical analyses were conducted using SPSS 13.0 (SPSS, Chicago, IL).

## Results

### Body weight curve, liver weight/body weight and WAT coefficient

**Fig 1:**
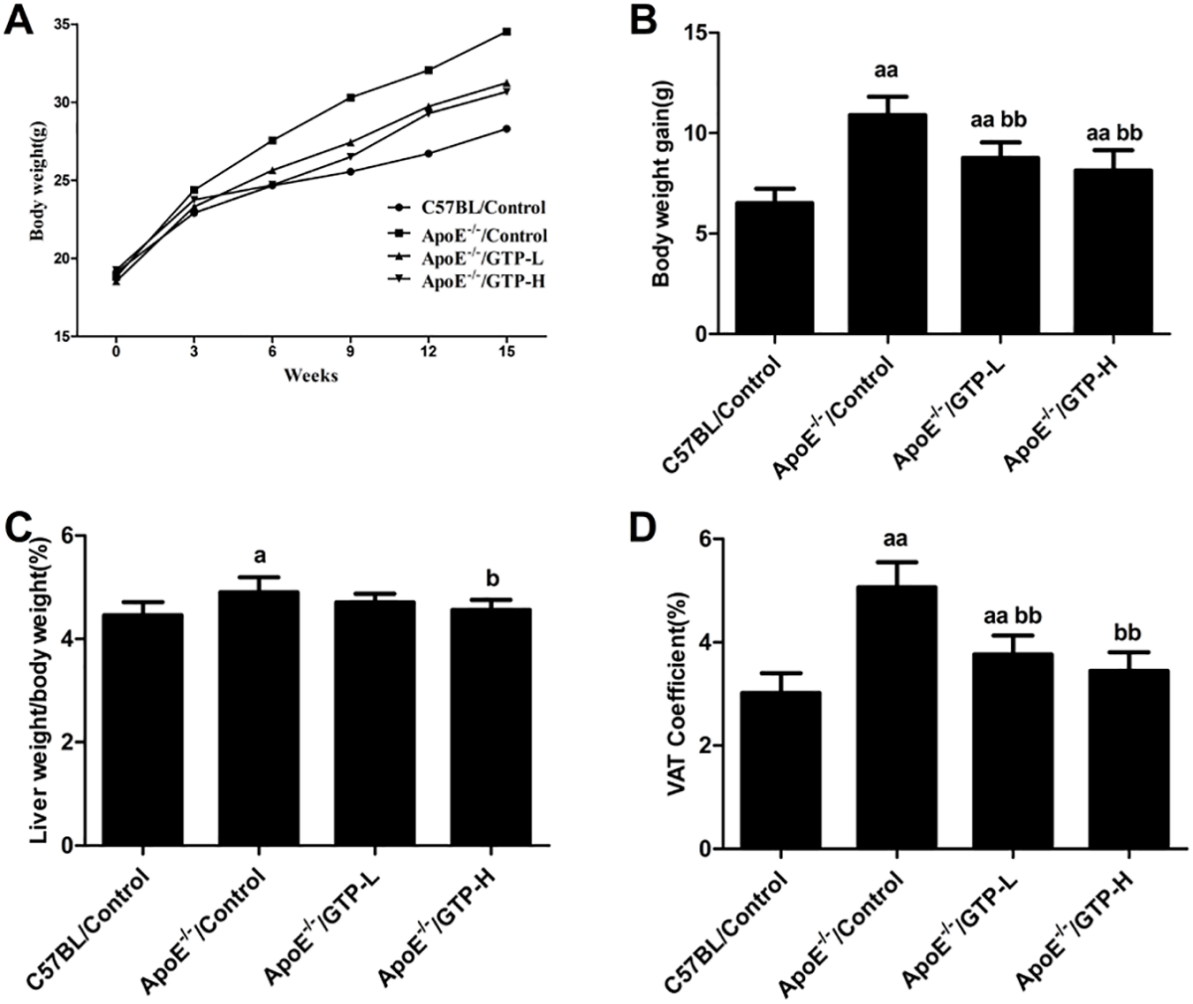
Effects of green tea polyphenol on body weight gain, liver weight/body weight and WAT coefficient in mice (n = 10 per group). (A) Body weight curve; (B) Body weight gain; (C) Liver weight/body weight; (D) WAT coefficient. ^a^, *P*<0.05 and ^aa^, *P*<0.01 compared to the C57BL/Control group; ^b^, *P*<0.05 and ^bb^, *P*<0.01 compared to the ApoE^-/-^/Control group. Data are expressed as the mean ± SD.

At baseline, no significant difference in body weight was observed among the four groups. Beginning in the third week, the body weight was increased in HFD-fed ApoE^-/-^ mice, and after GTP treatment, the body weights of the ApoE^-/-^/GTP-L group and the ApoE^-/-^/GTP-H group were decreased compared to the ApoE^-/-^/Control group (Fig 1A, Data in S1 Table). Additionally, the body weight gains of mice in the ApoE^-/-^/Control group were higher than those of the C57BL/Control group (*P*<0.01), and body weight gains were significantly decreased in mice of the ApoE^-/-^/GTP-L and ApoE^-/-^/GTP-H groups, compared with the ApoE^-/-^/Control group (*P*<0.01) (Fig 1B, data in S2 Table). Compared with mice in the C57BL/Control group, the liver weight/body weight of mice in the ApoE^-/-^/Control group obviously decreased (*P*<0.05); and the liver weight/body weight of mice in the ApoE^-/-^/GTP-H group was lower than that of the ApoE^-/-^/Control group (*P*<0.05) (Fig 1C, data in S3 Table). HFD markedly increased the WAT coefficient of mice in the ApoE^-/-^/Control group when compared to the C57BL/Control group (*P*<0.01). The WAT coefficient of mice in the ApoE^-/-^/GTP-L and ApoE^-/-^/GTP-H groups were significantly lower than that of the ApoE^-/-^/Control group (*P*<0.01) (Fig 1D, data in S4 Table).

### Serum and hepatic lipid parameters

**Fig 2:**
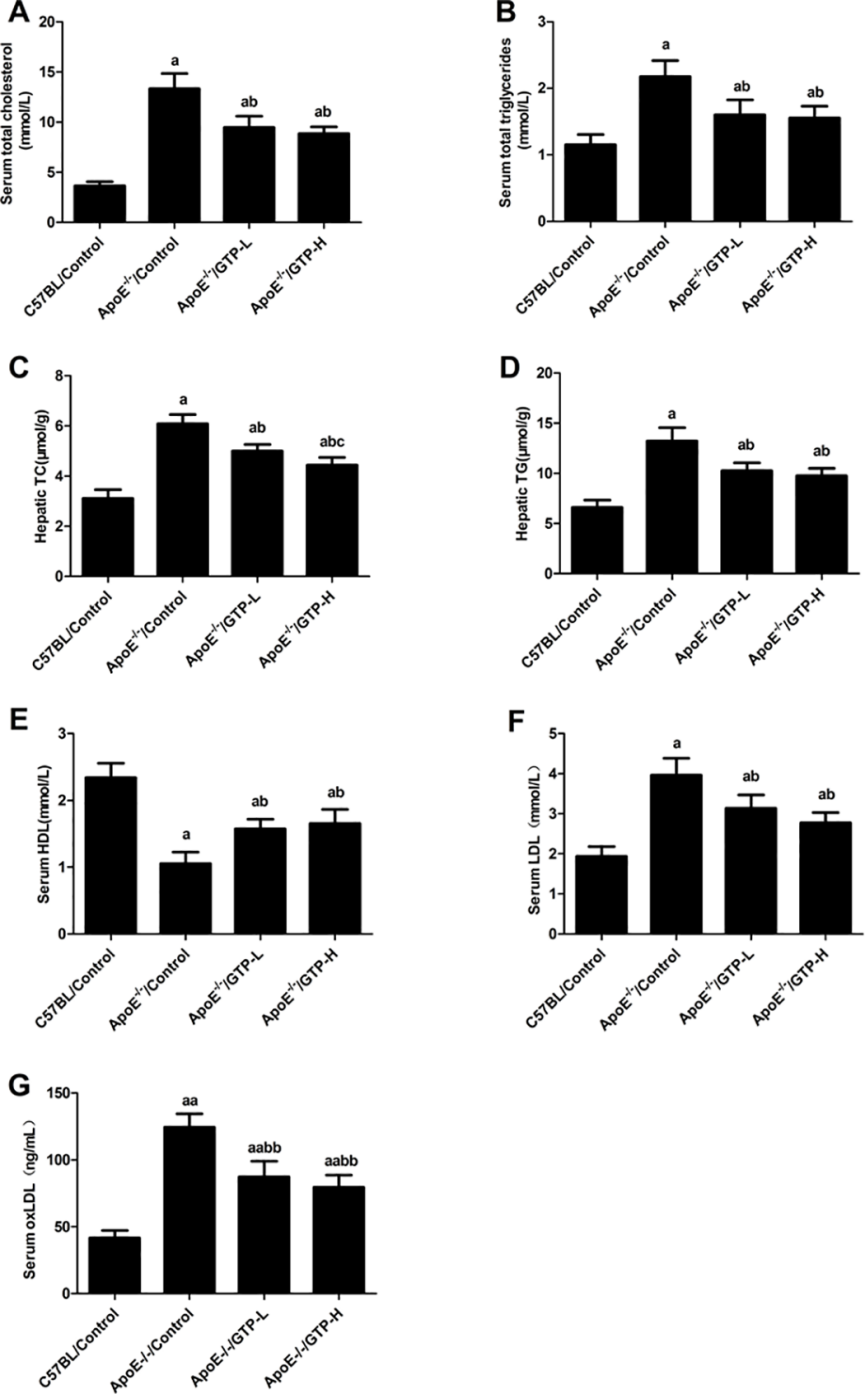
Effects of green tea polyphenol on serum and hepatic lipids in mice (n = 10 per group). (A) Serum total cholesterol; (B) Serum total triglyceride; (C) Hepatic cholesterol; (D) Hepatic triglycerides; (E) Serum HDL; (F) Serum LDL; (G) Serum oxLDL. ^a^ *P*<0.05 compared to C57BL/Control group; ^b^ *P*<0.05 compared to ApoE^-/-^/Control group; ^aa^ *P*<0.01 compared to C57BL/Control group; ^bb^ *P*<0.01 compared to ApoE^-/-^/Control group. Data are expressed as the mean ± SD.

After the 15-week treatment, total cholesterol and triglycerides in serum and livers of mice in the ApoE^-/-^/Control group were significantly higher when compared to the C57BL/Control group (*P*<0.05), and compared with the ApoE^-/-^/Control group, the ApoE^-/-^/GTP-L and ApoE^-/-^/GTP-H groups showed a significant decrease of total cholesterol and triglycerides in the liver (*P*<0.05) (Fig 2A–2D). Moreover, compared with the ApoE^-/-^/GTP-L group, the hepatic total cholesterol in the ApoE^-/-^/GTP-H group was markedly decreased (*P*<0.05) (Fig 2C). As shown in Fig 2E–2G, the HDL level in serum was significantly decreased in the ApoE^-/-^/Control group compared with the C57BL/Control group (*P*<0.05); in contrast, the LDL level and the oxLDL level in serum were significantly increased in the ApoE^-/-^/Control group compared to the C57BL/Control group (*P*<0.05 and *P*<0.01). Mice treated for 15 weeks with GTP had higher HDL in serum (*P*<0.05), lower LDL and oxLDL in serum (*P*<0.05 and *P*<0.01); in addition, there was no significant difference in HDL, LDL and oxLDL in the serum of the GTP-treated groups (Fig 2E–2G).

### Lipid droplets of the aorta and aortic lesion analysis

**Fig 3:**
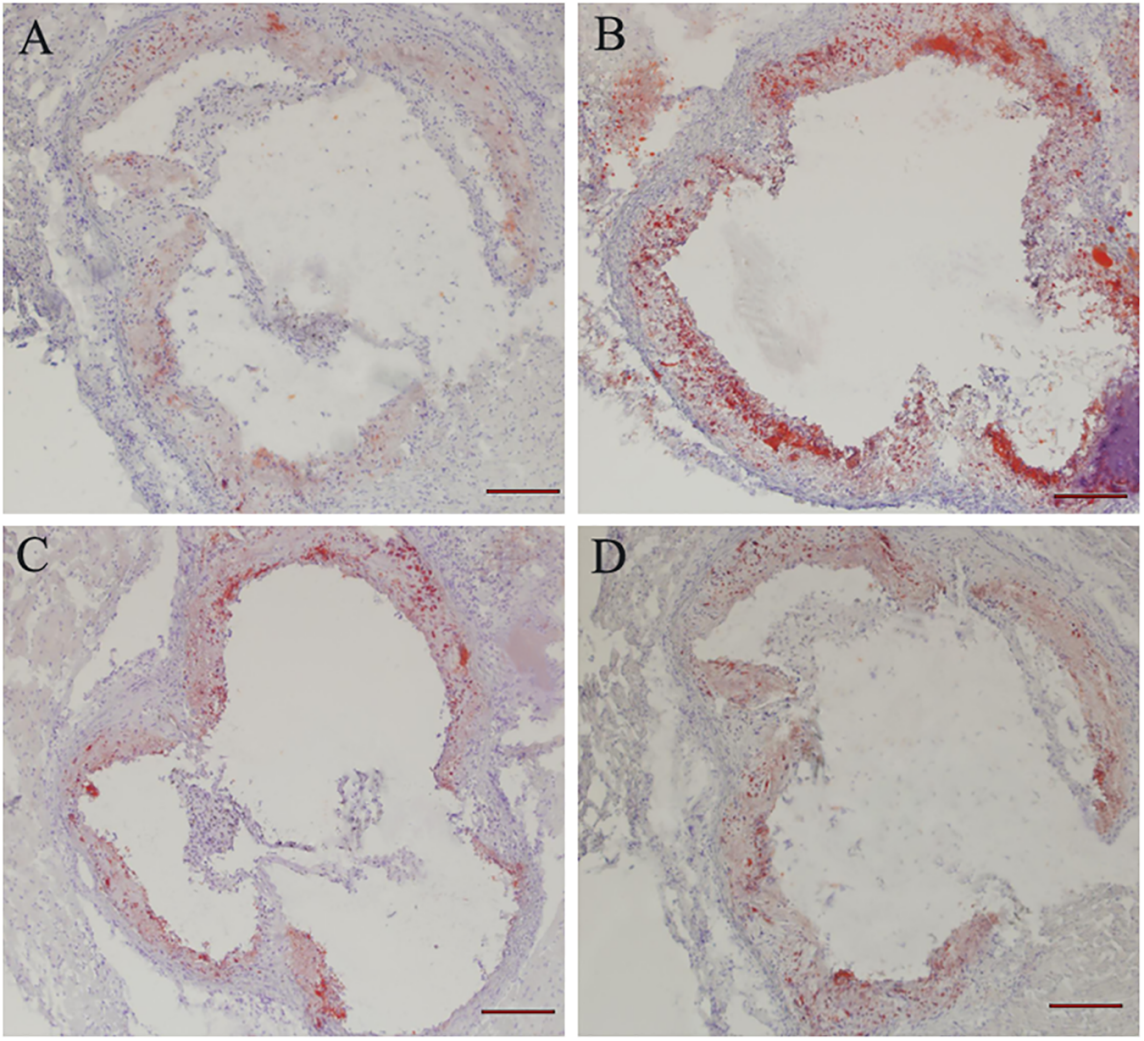
Oil red O staining of cross-sections of aortic roots in the hearts of ApoE^-/-^ mice (n = 3 per group). (A) C57BL/Control; (B) ApoE^-/-^/Control; (C) ApoE^-/-^/GTP-L; (D) ApoE^-/-^/GTP-H (20× magnification).

**Fig 4:**
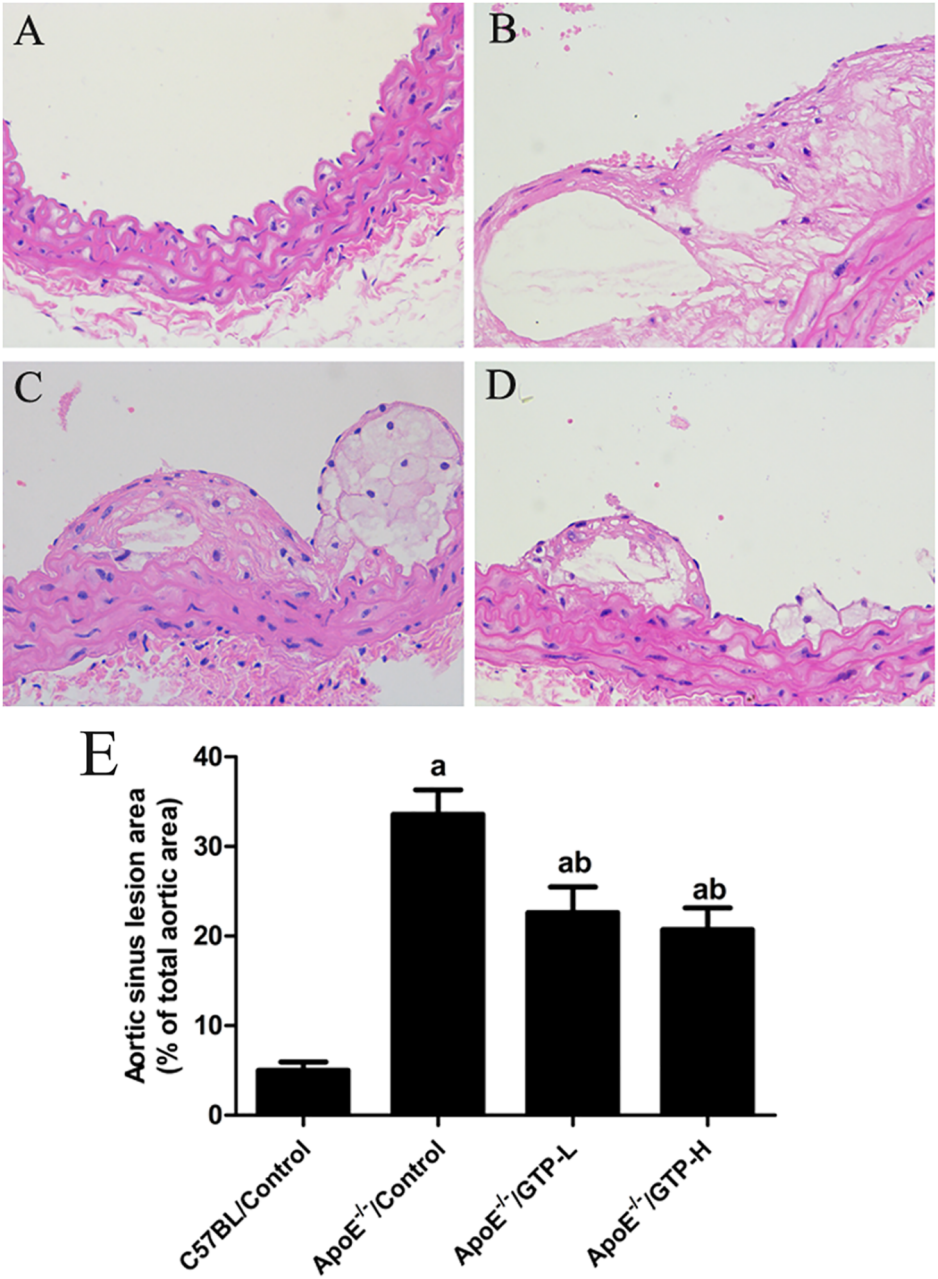
Aortic sinus lesion area of mice in the four groups (n = 3 per group). (A) C57BL/Control; (B) ApoE^-/-^/Control; (C) ApoE^-/-^/GTP-L; (D) ApoE^-/-^/GTP-H (40× magnification), representative aortic sinus sections from mice; (E) Quantitative analysis of the atherosclerotic lesions in aortic sinuses of mice. ^a^ *P*<0.05 compared to C57BL/Control group; ^b^ *P*<0.05 compared to ApoE^-/-^/Control group. Data are expressed as the mean ± SD.

As shown in Fig 3, ApoE^-/-^ mice fed with a HFD for 15 weeks showed a demonstrable increase in lipid droplets in the aorta. Moreover, long-term GTP treatment decreased the lipid droplets in the aorta of HFD-fed mice in the ApoE^-/-^/GTP-L and ApoE^-/-^/GTP-H groups. Furthermore, analysis of plaque area in the aortal roots (Fig 4) revealed a large number of vascular lesions in HFD-fed ApoE^-/-^ mice compared with STD-fed mice in the C57BL/Control group (*P*<0.05) (Fig 4). After the 15-week GTP treatment, plaque areas in the aortal roots of ApoE^-/-^ mice were significantly decreased (*P*<0.05) (Fig 4).

### Histology of liver

**Fig 5:**
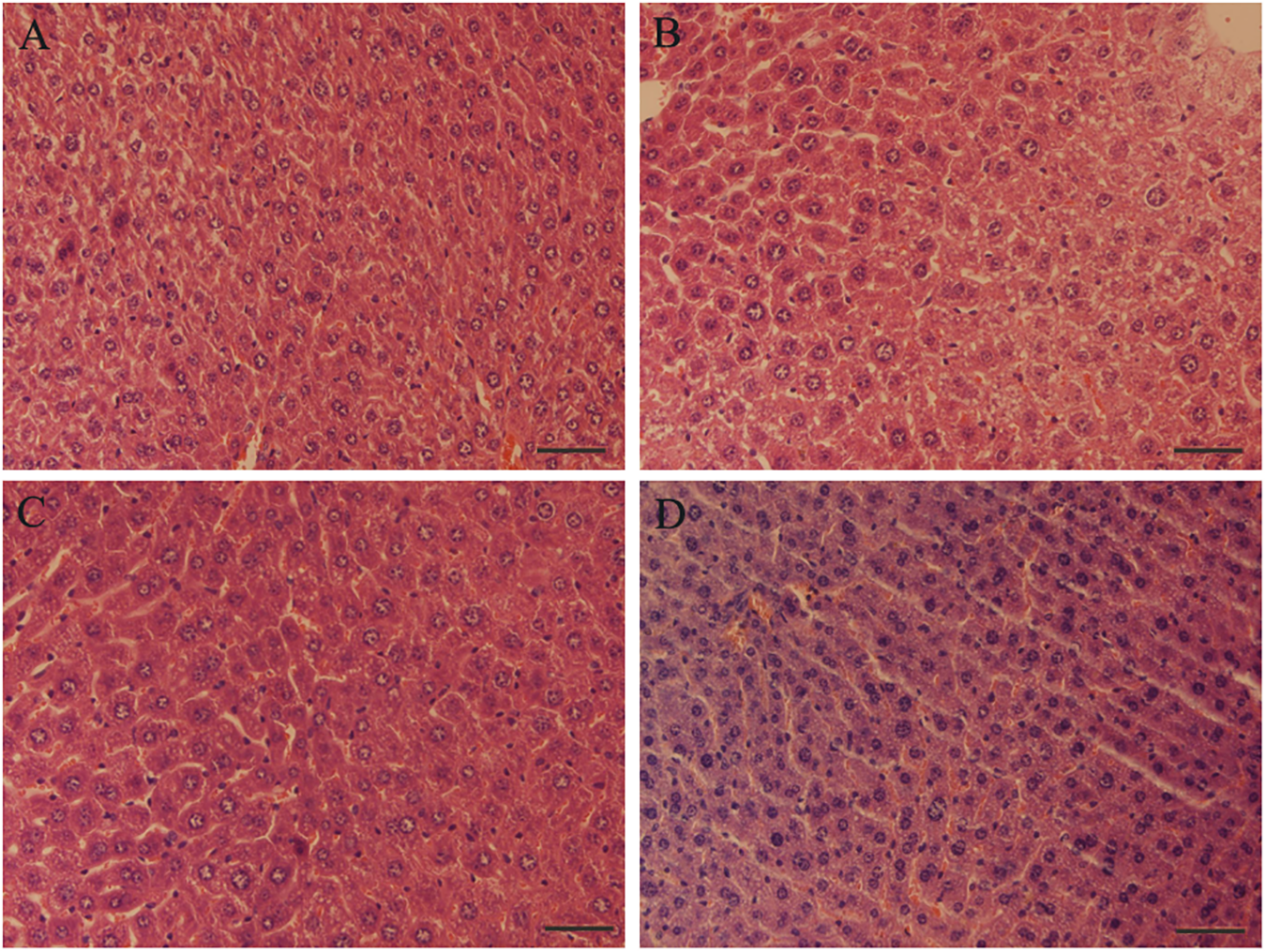
Hematoxylin and eosin (HE) staining of livers of mice in the four groups. (A) C57BL/Control; (B) ApoE^-/-^/Control; (C) ApoE^-/-^/GTP-L; (D) ApoE^-/-^/GTP-H (40× magnification, Bar = 100 μm), representative liver sections from mice.

The histological analysis of liver sections of mice in the four groups was completed by HE stain and subsequent light microscopy. Moderate centrilobular hepatic steatosis and microvesicular vacuolation (presence of many small lipid droplets) of livers in the ApoE^-/-^/Control group were observed compared to the C57BL/Control group (Fig 5A and 5B). After the 15-week treatment, the accumulation of widely scattered lipid droplets in the liver was significantly decreased in the ApoE^-/-^/GTP-L and ApoE^-/-^/GTP-H groups (Fig 5C and 5D).

### The mRNA expression in aorta and liver tissue in mice

**Fig 6:**
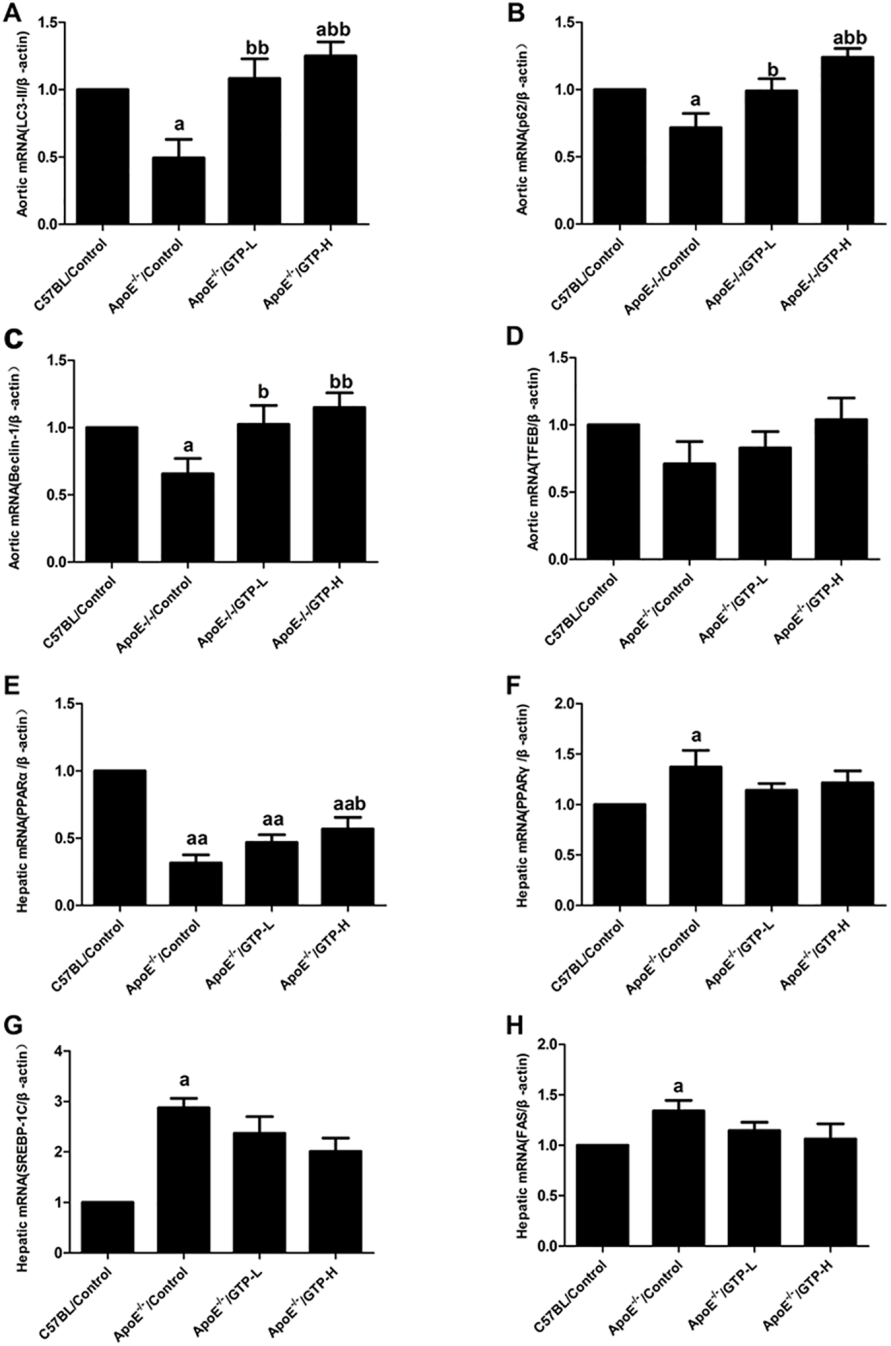
Effects of green tea polyphenol on mRNA expression in aortic and hepatic tissues in mice (n = 3). (A) *LC3*; (B) *p62*; (C) *Beclin-1*; (D) *TFEB*; (E) *PPARα*; (F) *PPARγ*; (G) *SREBP-1C*; (H) *FAS*. The mRNA of β-actin was quantified as an endogenous control. ^a^ *P*<0.05 and ^aa^ *P*<0.01 compared to C57BL/Control group; ^b^ *P*<0.05 and ^bb^ *P*<0.01 compared to ApoE^-/-^/Control group. Data are expressed as the mean ± SD.

As shown in Fig 6, a HFD markedly down-regulated mRNA expression of *LC3* in aorta tissue in ApoE^-/-^ mice compared with mice in the C57BL/Control group (*P*<0.05), whereas the expression was significantly increased by GTP treatment in the ApoE^-/-^/GTP-L and ApoE^-/-^/GTP-H groups (*P*<0.01). Compared to C57BL/Control group, the mRNA expression of *p62*/*SQSTM1* and *Beclin-1* were markedly decreased in aorta tissue in ApoE^-/-^ mice (*P*<0.05); and GTP treatment markedly decreased the mRNA expression of *p62*/*SQSTM1* and *Beclin-1* in aorta tissue in ApoE^-/-^ mice (*P*<0.05 and *P*<0.01). Moreover, no significant difference in the mRNA expression of *TFEB* in the aorta tissue of the four groups was observed. The mRNA expression of *PPARα*, an important regulator of hepatic lipid metabolism, was markedly decreased in HFD-fed ApoE^-/-^ mice compared with mice in the C57BL/Control group (*P*<0.01), and GTP treatment significantly up-regulated the mRNA expression of *PPARα* in mice of the ApoE^-/-^/GTP-H group compared with mice in the C57BL/Control group (*P*<0.05). The gene expressions of *SREBP-1C*, *PPARγ*, and *FAS* in livers of HFD-fed ApoE^-/-^ mice were significantly up-regulated compared to the C57BL/Control group (*P*<0.05); however, GTP treatment had no effect on the expression of these three genes (*SREBP-1C*, *PPARγ*, and *FAS*) in mice of the ApoE^-/-^/GTP-L and ApoE^-/-^/GTP-H groups (*P*>0.05).

### The protein level of LC3 and p62/SQSTM1 in aorta tissue and PPARα in liver tissue

**Fig 7:**
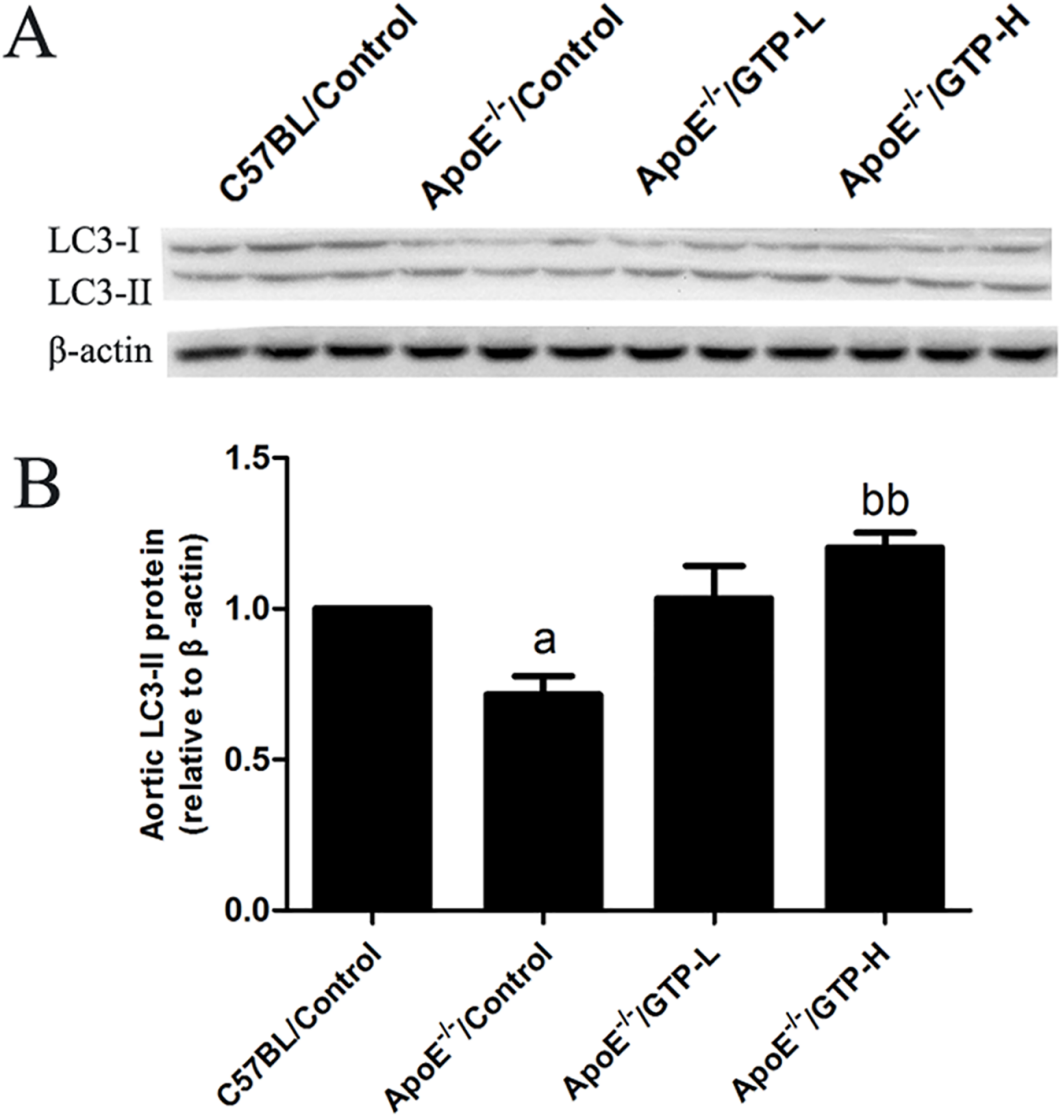
Effects of green tea polyphenol on protein expression of LC3 in aortic tissues of mice (n = 3). (A) Western blotting results for LC3 and β-actin. (B) Relative protein levels of LC3. Blotting with anti-β-actin was used as a protein loading control. ^a^ *P*<0.05 compared to C57BL/Control group; ^bb^ *P*<0.01 compared to ApoE^-/-^/Control group. Data are expressed as the mean ± SD.

**Fig 8:**
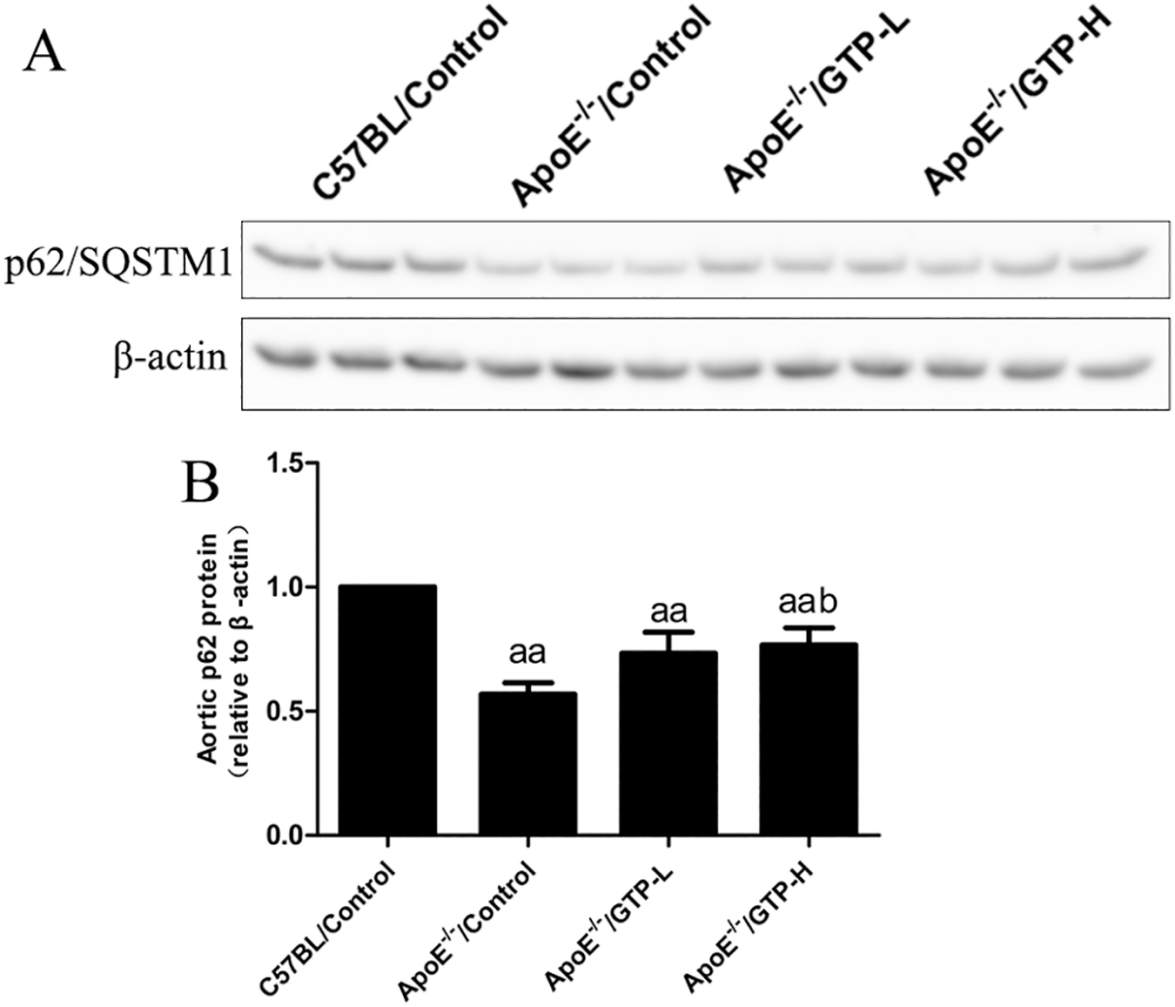
Effects of green tea polyphenol on protein expression of p62 in aortic tissues of mice (n = 3). (A) Western blotting results for p62 and β-actin. (B) Relative protein levels of p62. Blotting with anti-β-actin was used as a protein loading control. ^aa^ *P*<0.01 compared to C57BL/Control group; ^a^ *P*<0.05 compared to C57BL/Control group; ^b^ *P*<0.05 compared to ApoE^-/-^/Control group. Data are expressed as the mean ± SD.

**Fig 9:**
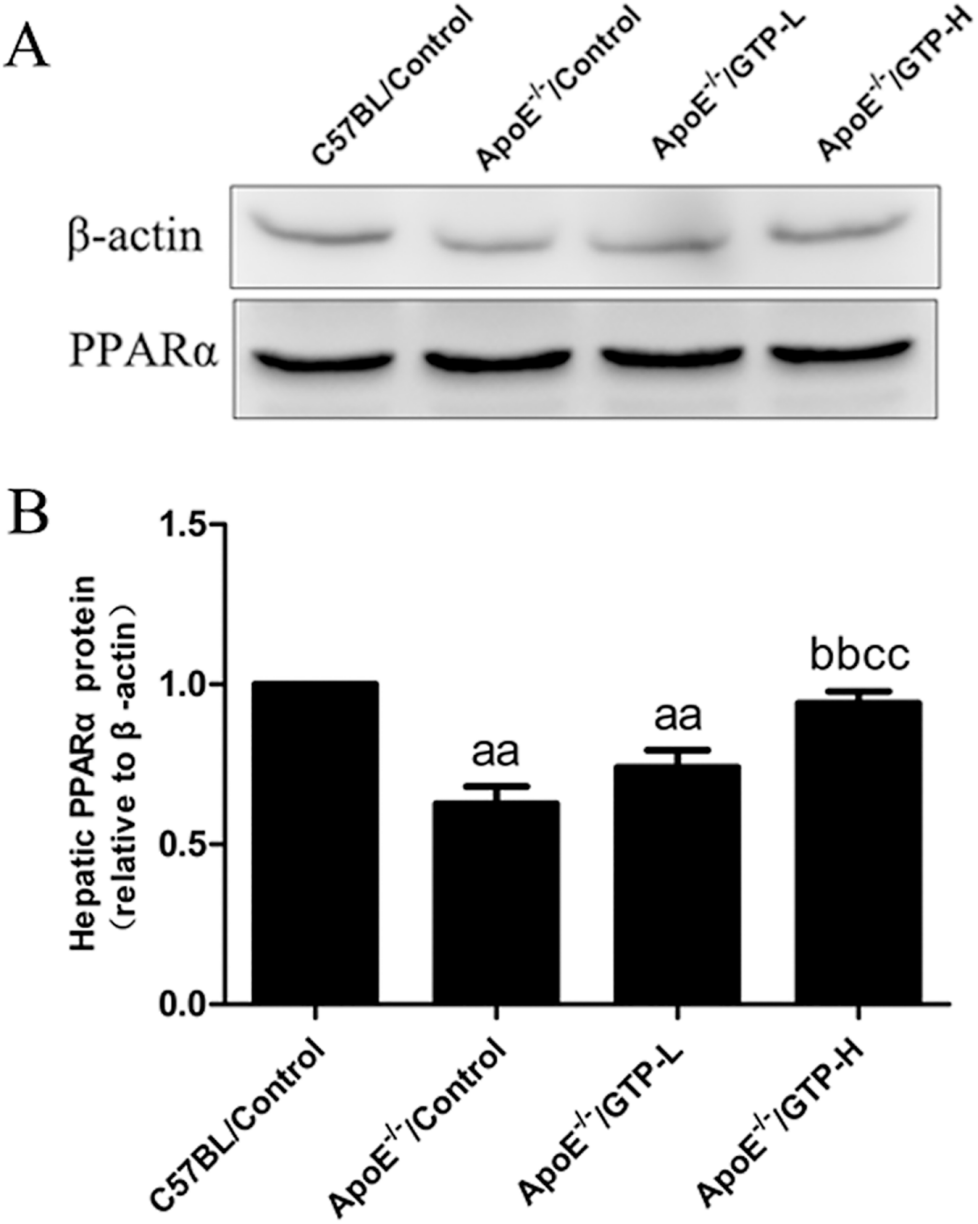
Effects of green tea polyphenol on protein expression of PPARα in hepatic tissues of mice (n = 3). Blotting with anti-β-actin was used as a protein loading control. ^aa^ *P*<0.01 compared to C57BL/Control group; ^bb^ *P*<0.01 compared to ApoE^-/-^/Control group; ^cc^ *P*<0.01 compared to ApoE^-/-^/GTP-L group. Data are expressed as the mean ± SD.

The protein expression of LC3 and p62/SQSTM1 in the aorta tissue (Figs 7 and 8) were evaluated by Western blot. The protein expression level of LC3 was decreased in HFD-fed ApoE^-/-^ mice compared to the C57BL/Control group (*P*<0.05). After the 15-week treatment of GTP, the protein expression levels of LC3 were significantly increased in mice of the ApoE^-/-^/GTP-H group (*P*<0.01). Moreover, compared to C57BL/Control group, the protein expression levels of p62/SQSTM1 was significantly decreased in HFD-fed ApoE^-/-^ mice (*P*<0.01). And compared to HFD-fed ApoE^-/-^ mice, the protein expression levels of p62/SQSTM1 was significantly increased in ApoE^-/-^/GTP-H group (*P*<0.05). To demonstrate the effect of GTP on hepatic metabolism in vivo, the protein expression of PPARα in liver was evaluated (Fig 9). The protein expression of PPARα in livers of mice in the ApoE^-/-^/Control group was significantly decreased compared to the C57BL/Control group (*P*<0.01). However, GTP treatment increased the protein expression of liver PPARα in the ApoE^-/-^/GTP-H group compared to the ApoE^-/-^/Control group (*P*<0.01) and the ApoE^-/-^/GTP-L group (*P*<0.01).

## Discussion

In the present study, we explored the effects of GTP, mainly consisting of EGCG and catechin, on atherosclerotic lesions and lipid metabolism in ApoE-knockout mice (an animal model of atherosclerosis). Our results have demonstrated that a 15-week GTP treatment improved the processing of atherosclerotic lesions, which may be involved in decreasing oxLDL in serum and up-regulating autophagy in the aorta, and alleviated lipid metabolism disorders via increasing hepatic PPARα expression. Furthermore, our results demonstrated that GTP treatment suppresses HFD-induced obesity, mainly through reducing the weight of white adipose tissue.

A previous study showed that 17 weeks of consumption of green tea leads to a reduction of atherosclerosis in rabbits [25]. Furthermore, Minatti J *et al*. demonstrated that administration of green tea extracts at low doses may contribute to a decrease in atherosclerosis progression in ApoE-knockout mice [26]. In accordance with the previous finding, we also observed the significant reduction of atherosclerotic lesion size in GTP-treated ApoE-knockout mice. Evidence indicates that oxLDL participates in the induction and the further development of atherosclerosis [27] and green tea has a protective effect on vascular endothelial cell-induced LDL oxidation[28]. In our study, we found that GTP could decrease the serum oxLDL levels. For further clarify the possible role of oxLDL and autophagy in GTP-treated ApoE-knockout mice, we determined the autophagy level in the aortas. Autophagy is a catabolic process that plays an important role in controlling central processes in cells by removing waste or excess proteins and organelles in animals [4]. In a previous study, it was shown that EGCG, a major polyphenol in green tea, regulates ectopic lipid accumulation through promoting autophagic flux, indicating that EGCG may be a potential therapeutic agent for the prevention of cardiovascular complications [10]. However, the prospective role of autophagy in the aorta or in the regulation of atherosclerosis remains poorly understood in vivo. In our study, decreased LC3-II, Beclin-1 in the aortas were observed in ApoE^-/-^/Control group, which indicated decreased autophagosomes in ApoE^-/-^/Control group; and GTP supplementation significantly increased LC3-II, Beclin-1. Moreover, p62/SQSTM1, an indicator of autophagosome degradation, was also decreased in ApoE^-/-^/Control group, while GTP supplementation increased p62/SQSTM1 mRNA and protein expression. The increase of p62/SQSTM1 protein levels in the aortas of ApoE^-/-^/GTP groups may be due to increased autophagosome flux to reduce atherosclerotic lesions formation. Previous studies reported that oxLDL actives mammalian target of rapamycin (mTOR) in THP-1cell [29], and an activator of mTOR inhibits oxLDL-induced autophagy and restricts atherosclerosis in ApoE-knockout mice [30]. From our results and those of others, we suggested that GTP could up-regulate autopahgy by inhibiting the levels of oxLDL in aortas of ApoE-knockout mice. All the above evidence showed that GTP supplementation could reduce oxLDL in serum, increase autophagy flux and lead to the reduction of atherosclerotic lesions and fatty acid accumulation in the aortas of ApoE-knockout mice.

Consistent with previous reports, our results showed that a HFD led to an increase in body weight gain as well as an increase in the white fat content and that GTP decreased body weight, body weight gain, liver weight/body weight ratio and WAT coefficient in HFD-fed ApoE-knockout mice [31, 32]. Based on these parameters, we demonstrated the function of GTP in reducing body weight and WAT coefficient, mainly through reducing fat content in mice. Modern lifestyle changes and the Western-dietary model have contributed to the prevalence of dyslipidemia, hepatic steatosis and atherosclerosis. In our study, a HFD resulted in lipid metabolic disorder and atherosclerosis in ApoE-knockout mice. Corresponding with the dyslipidemia, we further observed an increased fatty ifiltration into hepatocytes, which would have resulted in hepatic lipid accumulation and steatosis, in ApoE-knockout mice when compared to the mice in the C57BL/Control group. Dyslipidemia and hepatic steatosis were recognized as the early events of atherosclerosis; after 15-week supplementation, GTP improved the dyslipidemia and hepatic steatosis in ApoE-knockout mice.

SREBP-1C is a key gene transcription factor for fatty acid synthesis and lipid metabolism [33], and activated SREBP-1C can regulate the lipogenic enzymes of FAS to inhibit lipogenesis in liver tissues [34]. In our study, HFD markedly changed the mRNA expressions of hepatic SREBP-1C and FAS levels. It has recently been reported that EGCG (50 mg/kg/day) treatment for 16 weeks did not affect the protein expression of SREBP-1C and decreased the protein expression of FAS in the livers of HFD-fed Swiss mice [32]. However, in the present study, no changes in the mRNA expression of SREBP-1C and FAS in liver were observed in GTP-treated ApoE-knockout mice. In addition, PPARα and PPARγ are the members of the nuclear receptor super family PPARs that are the critical regulators of adipogenesis and may be related to hepatic lipid metabolism disorders [35, 36]. Similar to previous studies, we observed that a HFD resulted in lipid metabolic disorder, and GTP treatment alleviated dyslipidemia and reduced fat deposited in the liver by increasing the mRNA and protein expressions of hepatic PPARα in HFD-fed animals [20]. These results confirmed that GTP exerts strong lipid-lowering effects, and its prevention of hepatic fatty deposition via regulating the expression of lipid metabolic regulator PPARα could contribute to anti-atherosclerosis. In our previous study, we observed a significant increase in expression of PPARγ in the adipose tissue of HFD-fed Wistar rats [18]. However, the mRNA expression of hepatic PPARγ did not change due to GTP treatment in HFD-fed ApoE-knockout mice. These results indicate that PPARγ may play a critical role in fat reduction in adipose tissues rather than liver tissues.

In summary, GTP decreases oxLDL levels in serum, up-regulates autophagy levels of aorta tissues, which contributes to a reduction of HFD-induced accumulation of lipid droplets in the endothelial cells of ApoE-knockout mice. In addition, long-term GTP ingestion markedly alleviates lipid metabolism disorders induced by a high-fat diet, which may be related to the up-regulation of hepatic PPARα expression. These effects of GTP could be a benefit for preventing lipid metabolic disorders and atherosclerosis. GTP acts multi-factorially, not only by decreasing lipid droplets in endothelial cells but also by regulating lipid metabolism disorders in the liver and reducing white adipose tissue.

## Supporting information

S1 Table Effects of green tea polyphenol on body weight. (DOC)

S2 Table Effects of green tea polyphenol on body weight gain. (DOC)

S3 Table Effects of green tea polyphenol on liver weight and body weight ratio. (DOC)

S4 Table Effects of green tea polyphenol on VAT coefficient. (DOC)

S5 Table Effects of green tea polyphenol on serum total cholesterol. (DOC)

S6 Table Effects of green tea polyphenol on serum total triglycerides. (DOC)

S7 Table Effects of green tea polyphenol on hepatic TC. (DOC)

S8 Table Effects of green tea polyphenol on hepatic TG. (DOC)

S9 Table Effects of green tea polyphenol on serum HDL. (DOC)

S10 Table Effects of green tea polyphenol on serum LDL. (DOC)

S11 Table Atherosclerotic lesion analysis. (DOC)

S12 Table Effects of green tea polyphenol on mRNA expressions. (DOC)

S13 Table Effects of green tea polyphenol on protein expressions. (DOC)

S14 Table Effects of green tea polyphenol on serum oxLDL (DOC)

S1 Fig Effects of green tea polyphenol on LC3 and beta-actin protein levels in the aorta. (TIF)

S2 Fig Effects of green tea polyphenol on p62 protein levels in the aorta. (TIF)

S3 Fig Effects of green tea polyphenol on beta-actin protein levels (control of p62) in the aorta. (TIF)

S4 Fig Effects of green tea polyphenol on PPAR-alpha protein levels in liver. (TIF)

S5 Fig Effects of green tea polyphenol on beta-actin protein levels (control of PPAR-alpha) in liver. (TIF)

## Abbreviations

GTP: Green tea polyphenol
HFD: high-fat diet
EGCG: Epigallocatechin-3-gallate
LC3: microtubule-associated protein 1A/1B-light chain 3
TFEB: the transcription factor EB
PPARs: peroxisome proliferator-activated receptors
SREBP-1C: the transcriptional factor sterol regulatory element-binding protein-1C
FAS: fatty acid synthase
oxLDL: oxidized low-density lipoprotein cholesterol

## References

[1] Ross R. Atherosclerosis is an inflammatory disease. American heart journal. 1999;138(5 Pt 2):S419–20. Epub 1999/10/28. . 10539839 10.1016/s0002-8703(99)70266-8

[2] Rioufol G, Finet G, Ginon I, Andre-Fouet X, Rossi R, Vialle E, et al Multiple atherosclerotic plaque rupture in acute coronary syndrome: a three-vessel intravascular ultrasound study. Circulation. 2002;106(7):804–8. Epub 2002/08/15. . 12176951 10.1161/01.cir.0000025609.13806.31

[3] Sima AV, Stancu CS, Simionescu M. Vascular endothelium in atherosclerosis. Cell and tissue research. 2009;335(1):191–203. Epub 2008/09/18. doi: 10.1007/s00441-008-0678-5 . 18797930 10.1007/s00441-008-0678-5

[4] Sridhar S, Botbol Y, Macian F, Cuervo AM. Autophagy and disease: always two sides to a problem. The Journal of pathology. 2012;226(2):255–73. Epub 2011/10/13. doi: 10.1002/path.3025 ; PubMed Central PMCID: PMC3996449. 21990109 10.1002/path.3025 PMC3996449

[5] Vindis C. Autophagy: an emerging therapeutic target in vascular diseases. British journal of pharmacology. 2015;172(9):2167–78. Epub 2014/12/30. doi: 10.1111/bph.13052 ; PubMed Central PMCID: PMC4403086. 25537552 10.1111/bph.13052 PMC4403086

[6] Luo Y, Lu S, Zhou P, Ai QD, Sun GB, Sun XB. Autophagy: An Exposing Therapeutic Target in Atherosclerosis. Journal of cardiovascular pharmacology. 2016;67(3):266–74. Epub 2015/11/19. doi: 10.1097/FJC.0000000000000342 . 26580134 10.1097/FJC.0000000000000342

[7] Pang J, Zhang Z, Zheng TZ, Bassig BA, Mao C, Liu X, et al Green tea consumption and risk of cardiovascular and ischemic related diseases: A meta-analysis. International journal of cardiology. 2016;202:967–74. Epub 2015/09/01. doi: 10.1016/j.ijcard.2014.12.176 . 26318390 10.1016/j.ijcard.2014.12.176

[8] Wolfram S. Effects of green tea and EGCG on cardiovascular and metabolic health. Journal of the American College of Nutrition. 2007;26(4):373S–88S. Epub 2007/10/02. . 17906191 10.1080/07315724.2007.10719626

[9] Wang ZM, Zhou B, Wang YS, Gong QY, Wang QM, Yan JJ, et al Black and green tea consumption and the risk of coronary artery disease: a meta-analysis. The American journal of clinical nutrition. 2011;93(3):506–15. Epub 2011/01/21. doi: 10.3945/ajcn.110.005363 . 21248184 10.3945/ajcn.110.005363

[10] Kim HS, Montana V, Jang HJ, Parpura V, Kim JA. Epigallocatechin gallate (EGCG) stimulates autophagy in vascular endothelial cells: a potential role for reducing lipid accumulation. The Journal of biological chemistry. 2013;288(31):22693–705. Epub 2013/06/12. doi: 10.1074/jbc.M113.477505 ; PubMed Central PMCID: PMC3829354. 23754277 10.1074/jbc.M113.477505 PMC3829354

[11] Yamagata K, Xie Y, Suzuki S, Tagami M. Epigallocatechin-3-gallate inhibits VCAM-1 expression and apoptosis induction associated with LC3 expressions in TNFalpha-stimulated human endothelial cells. Phytomedicine: international journal of phytotherapy and phytopharmacology. 2015;22(4):431–7. Epub 2015/05/01. doi: 10.1016/j.phymed.2015.01.011 . 25925964 10.1016/j.phymed.2015.01.011

[12] Cai Y, Kurita-Ochiai T, Hashizume T, Yamamoto M. Green tea epigallocatechin-3-gallate attenuates Porphyromonas gingivalis-induced atherosclerosis. Pathogens and disease. 2013;67(1):76–83. Epub 2013/04/27. doi: 10.1111/2049-632X.12001 . 23620122 10.1111/2049-632X.12001

[13] Chyu KY, Babbidge SM, Zhao X, Dandillaya R, Rietveld AG, Yano J, et al Differential effects of green tea-derived catechin on developing versus established atherosclerosis in apolipoprotein E-null mice. Circulation. 2004;109(20):2448–53. Epub 2004/05/12. doi: 10.1161/01.CIR.0000128034.70732.C2 . 15136500 10.1161/01.CIR.0000128034.70732.C2

[14] Li J, Sapper TN, Mah E, Rudraiah S, Schill KE, Chitchumroonchokchai C, et al Green tea extract provides extensive Nrf2-independent protection against lipid accumulation and NFkappaB pro- inflammatory responses during nonalcoholic steatohepatitis in mice fed a high-fat diet. Molecular nutrition & food research. 2016;60(4):858–70. Epub 2015/12/19. doi: 10.1002/mnfr.201500814 ; PubMed Central PMCID: PMC4828297. 26679056 10.1002/mnfr.201500814 PMC4828297

[15] Onakpoya I, Spencer E, Heneghan C, Thompson M. The effect of green tea on blood pressure and lipid profile: a systematic review and meta-analysis of randomized clinical trials. Nutrition, metabolism, and cardiovascular diseases: NMCD. 2014;24(8):823–36. Epub 2014/03/29. doi: 10.1016/j.numecd.2014.01.016 . 24675010 10.1016/j.numecd.2014.01.016

[16] Ivanova D, Vankova D, Nashar M. Agrimonia eupatoria tea consumption in relation to markers of inflammation, oxidative status and lipid metabolism in healthy subjects. Archives of physiology and biochemistry. 2013;119(1):32–7. Epub 2012/10/20. doi: 10.3109/13813455.2012.729844 . 23078582 10.3109/13813455.2012.729844

[17] Santamarina AB, Carvalho-Silva M, Gomes LM, Okuda MH, Santana AA, Streck EL, et al Decaffeinated green tea extract rich in epigallocatechin-3-gallate prevents fatty liver disease by increased activities of mitochondrial respiratory chain complexes in diet-induced obesity mice. The Journal of nutritional biochemistry. 2015;26(11):1348–56. Epub 2015/08/25. doi: 10.1016/j.jnutbio.2015.07.002 . 26300331 10.1016/j.jnutbio.2015.07.002

[18] Tian C, Ye X, Zhang R, Long J, Ren W, Ding S, et al Green tea polyphenols reduced fat deposits in high fat-fed rats via erk1/2-PPARgamma-adiponectin pathway. PloS one. 2013;8(1):e53796 Epub 2013/01/24. doi: 10.1371/journal.pone.0053796 ; PubMed Central PMCID: PMC3546082. 23342006 10.1371/journal.pone.0053796 PMC3546082

[19] Millar JS. Novel benefits of peroxisome proliferator-activated receptors on cardiovascular risk. Current opinion in lipidology. 2013;24(3):233–8. Epub 2013/04/19. doi: 10.1097/MOL.0b013e3283613a7d . 23594710 10.1097/MOL.0b013e3283613a7d

[20] Lee SJ, Jia Y. The effect of bioactive compounds in tea on lipid metabolism and obesity through regulation of peroxisome proliferator-activated receptors. Current opinion in lipidology. 2015;26(1):3–9. Epub 2015/01/01. doi: 10.1097/MOL.0000000000000145 . 25551797 10.1097/MOL.0000000000000145

[21] Ding S, Zuo X, Fan Y, Li H, Zhao N, Yang H, et al Environmentally Relevant Dose of Bisphenol A Does Not Affect Lipid Metabolism and Has No Synergetic or Antagonistic Effects on Genistein's Beneficial Roles on Lipid Metabolism. PloS one. 2016;11(5):e0155352 Epub 2016/05/14. doi: 10.1371/journal.pone.0155352 ; PubMed Central PMCID: PMC4865196. 27171397 10.1371/journal.pone.0155352 PMC4865196

[22] Lin J, Yang R, Tarr PT, Wu PH, Handschin C, Li S, et al Hyperlipidemic effects of dietary saturated fats mediated through PGC-1beta coactivation of SREBP. Cell. 2005;120(2):261–73. Epub 2005/02/01. doi: 10.1016/j.cell.2004.11.043 . 15680331 10.1016/j.cell.2004.11.043

[23] Martinez N, Urpi-Sarda M, Martinez-Gonzalez MA, Andres-Lacueva C, Mitjavila MT. Dealcoholised beers reduce atherosclerosis and expression of adhesion molecules in apoE-deficient mice. The British journal of nutrition. 2011;105(5):721–30. Epub 2010/12/08. doi: 10.1017/S0007114510004289 . 21134332 10.1017/S0007114510004289

[24] Ying CJ, Xu JW, Ikeda K, Takahashi K, Nara Y, Yamori Y. Tea polyphenols regulate nicotinamide adenine dinucleotide phosphate oxidase subunit expression and ameliorate angiotensin II-induced hyperpermeability in endothelial cells. Hypertension research: official journal of the Japanese Society of Hypertension. 2003;26(10):823–8. Epub 2003/11/19. . 14621186 10.1291/hypres.26.823

[25] Kavantzas N, Chatziioannou A, Yanni AE, Tsakayannis D, Balafoutas D, Agrogiannis G, et al Effect of green tea on angiogenesis and severity of atherosclerosis in cholesterol-fed rabbit. Vascular pharmacology. 2006;44(6):461–3. Epub 2006/05/16. doi: 10.1016/j.vph.2006.03.008 . 16697267 10.1016/j.vph.2006.03.008

[26] Minatti J, Wazlawik E, Hort MA, Zaleski FL, Ribeiro-do-Valle RM, Maraschin M, et al Green tea extract reverses endothelial dysfunction and reduces atherosclerosis progression in homozygous knockout low-density lipoprotein receptor mice. Nutr Res. 2012;32(9):684–93. Epub 2012/10/23. doi: 10.1016/j.nutres.2012.08.003 . 23084641 10.1016/j.nutres.2012.08.003

[27] Lusis AJ. Atherosclerosis. Nature. 2000;407(6801):233–41. Epub 2000/09/23. doi: 10.1038/35025203 ; PubMed Central PMCID: PMC2826222. 11001066 10.1038/35025203 PMC2826222

[28] Yang TT, Koo MW. Inhibitory effect of Chinese green tea on endothelial cell-induced LDL oxidation. Atherosclerosis. 2000;148(1):67–73. Epub 1999/12/02. . 10580172 10.1016/s0021-9150(99)00239-7

[29] Zabirnyk O, Liu W, Khalil S, Sharma A, Phang JM. Oxidized low-density lipoproteins upregulate proline oxidase to initiate ROS-dependent autophagy. Carcinogenesis. 2010;31(3):446–54. Epub 2009/11/28. doi: 10.1093/carcin/bgp299 ; PubMed Central PMCID: PMC2832543. 19942609 10.1093/carcin/bgp299 PMC2832543

[30] Peng N, Meng N, Wang S, Zhao F, Zhao J, Su L, et al An activator of mTOR inhibits oxLDL-induced autophagy and apoptosis in vascular endothelial cells and restricts atherosclerosis in apolipoprotein E(-)/(-) mice. Scientific reports. 2014;4:5519 Epub 2014/07/02. doi: 10.1038/srep05519 ; PubMed Central PMCID: PMC4076681. 24980430 10.1038/srep05519 PMC4076681

[31] Lee LS, Choi JH, Sung MJ, Hur JY, Hur HJ, Park JD, et al Green tea changes serum and liver metabolomic profiles in mice with high-fat diet-induced obesity. Molecular nutrition & food research. 2015;59(4):784–94. Epub 2015/01/30. doi: 10.1002/mnfr.201400470 . 25631872 10.1002/mnfr.201400470

[32] Santamarina AB, Oliveira JL, Silva FP, Carnier J, Mennitti LV, Santana AA, et al Green Tea Extract Rich in Epigallocatechin-3-Gallate Prevents Fatty Liver by AMPK Activation via LKB1 in Mice Fed a High-Fat Diet. PloS one. 2015;10(11):e0141227 Epub 2015/11/05. doi: 10.1371/journal.pone.0141227 ; PubMed Central PMCID: PMC4633218. 26536464 10.1371/journal.pone.0141227 PMC4633218

[33] Horton JD, Goldstein JL, Brown MS. SREBPs: activators of the complete program of cholesterol and fatty acid synthesis in the liver. The Journal of clinical investigation. 2002;109(9):1125–31. Epub 2002/05/08. doi: 10.1172/JCI15593 ; PubMed Central PMCID: PMC150968. 11994399 10.1172/JCI15593 PMC150968

[34] Sato R. Sterol metabolism and SREBP activation. Archives of biochemistry and biophysics. 2010;501(2):177–81. Epub 2010/06/15. doi: 10.1016/j.abb.2010.06.004 . 20541520 10.1016/j.abb.2010.06.004

[35] Kersten S. Integrated physiology and systems biology of PPARalpha. Molecular metabolism. 2014;3(4):354–71. Epub 2014/06/20. doi: 10.1016/j.molmet.2014.02.002 ; PubMed Central PMCID: PMC4060217. 24944896 10.1016/j.molmet.2014.02.002 PMC4060217

[36] Rosen ED, Spiegelman BM. PPARgamma: a nuclear regulator of metabolism, differentiation, and cell growth. The Journal of biological chemistry. 2001;276(41):37731–4. Epub 2001/07/19. doi: 10.1074/jbc.R100034200 . 11459852 10.1074/jbc.R100034200

