# Supplementary material for: Green tea polyphenol treatment attenuates atherosclerosis in high-fat diet-fed apolipoprotein E-knockout mice via alleviating dyslipidemia and up-regulating autophagy
Source: PLoS One. 2017 Aug 4;12(8):e0181666. doi: 10.1371/journal.pone.0181666 (PMC5544182; doi:10.1371/journal.pone.0181666)
Supplement: S1 Table — (DOC) [file pone.0181666.s001.doc]

**S1 Table. Effects of green tea polyphenol on body weight** (Mean)

| Week | C57BL/6J/Control group | ApoE^-/-^/Control group | ApoE^-/-^/GTP-L group | ApoE^-/-^/ GTP-H group |
| --- | --- | --- | --- | --- |
| 0 | 19.11 | 18.83 | 18.55 | 19.25 |
| 3 | 22.91 | 24.39 | 23.31 | 23.75 |
| 6 | 24.67 | 27.55 | 25.65 | 24.70 |
| 9 | 25.55 | 30.29 | 27.43 | 26.50 |
| 12 | 26.72 | 32.04 | 29.73 | 29.28 |
| 15 | 28.30 | 34.53 | 31.25 | 30.67 |
