# Supplementary material for: Green tea polyphenol treatment attenuates atherosclerosis in high-fat diet-fed apolipoprotein E-knockout mice via alleviating dyslipidemia and up-regulating autophagy
Source: PLoS One. 2017 Aug 4;12(8):e0181666. doi: 10.1371/journal.pone.0181666 (PMC5544182; doi:10.1371/journal.pone.0181666)
Supplement: S2 Table — (DOC) [file pone.0181666.s002.doc]

**S2 Table. Effects of green tea polyphenol on body weight gain**

|  | C57BL/6J/Control group | ApoE^-/-^/Control group | ApoE^-/-^/GTP-L group | ApoE^-/-^/ GTP-H group |
| --- | --- | --- | --- | --- |
| Mean | 5.92 | 10.90 | 8.77 | 8.13 |
| SD | 1.36 | 0.91 | 0.77 | 1.03 |
