# Supplementary material for: Green tea polyphenol treatment attenuates atherosclerosis in high-fat diet-fed apolipoprotein E-knockout mice via alleviating dyslipidemia and up-regulating autophagy
Source: PLoS One. 2017 Aug 4;12(8):e0181666. doi: 10.1371/journal.pone.0181666 (PMC5544182; doi:10.1371/journal.pone.0181666)
Supplement: S4 Table — (DOC) [file pone.0181666.s004.doc]

**S4 Table. Effects of green tea polyphenol on VAT coefficient**

|  | C57BL/6J/Control group | ApoE^-/-^/Control group | ApoE^-/-^/GTP-L group | ApoE^-/-^/ GTP-H group |
| --- | --- | --- | --- | --- |
| Mean | 3.09 | 5.08 | 4.03 | 3.59 |
| SD | 0.43 | 0.46 | 0.40 | 0.35 |
