# Supplementary material for: Green tea polyphenol treatment attenuates atherosclerosis in high-fat diet-fed apolipoprotein E-knockout mice via alleviating dyslipidemia and up-regulating autophagy
Source: PLoS One. 2017 Aug 4;12(8):e0181666. doi: 10.1371/journal.pone.0181666 (PMC5544182; doi:10.1371/journal.pone.0181666)
Supplement: S6 Table — (DOC) [file pone.0181666.s006.doc]

**S6 Table. Effects of green tea polyphenol on serum total triglycerides**

|  | C57BL/6J/Control group | ApoE^-/-^/Control group | ApoE^-/-^/GTP-L group | ApoE^-/-^/ GTP-H group |
| --- | --- | --- | --- | --- |
| Mean | 1.15 | 2.17 | 1.60 | 1.56 |
| SD | 0.15 | 0.24 | 0.23 | 0.18 |
