# Supplementary material for: Green tea polyphenol treatment attenuates atherosclerosis in high-fat diet-fed apolipoprotein E-knockout mice via alleviating dyslipidemia and up-regulating autophagy
Source: PLoS One. 2017 Aug 4;12(8):e0181666. doi: 10.1371/journal.pone.0181666 (PMC5544182; doi:10.1371/journal.pone.0181666)
Supplement: S12 Table — (DOC) [file pone.0181666.s012.doc]

**S12 Table. Effects of green tea polyphenol on mRNA expressions**

|  | C57BL/6J/Control group | ApoE^-/-^/Control group | ApoE^-/-^/GTP-L group | ApoE^-/-^/ GTP-H group |
| --- | --- | --- | --- | --- |
| *LC3* | 1.00 | 0.60 | 0.92 | 1.32 |
|  | 1.00 | 0.44 | 1.2 | 1.19 |
|  | 1.00 | 0.39 | 1.13 | 1.43 |
| *p62* | 1.00 | 0.82 | 1.08 | 1.23 |
|  | 1.00 | 0.72 | 0.99 | 1.18 |
|  | 1.00 | 0.61 | 0.90 | 1.31 |
| *Beclin-1* | 1.00 | 0.69 | 0.89 | 1.24 |
|  | 1.00 | 0.53 | 1.17 | 1.18 |
|  | 1.00 | 0.75 | 1.01 | 1.03 |
| *TFEB* | 1.00 | 0.56 | 0.95 | 1.02 |
|  | 1.00 | 0.68 | 0.83 | 0.89 |
|  | 1.00 | 0.89 | 0.71 | 1.21 |
| *PPARα* | 1.00 | 0.34 | 0.48 | 0.56 |
|  | 1.00 | 0.25 | 0.41 | 0.66 |
|  | 1.00 | 0.36 | 0.52 | 0.49 |
| *PPARγ* | 1.00 | 1.34 | 1.08 | 1.2 |
|  | 1.00 | 1.55 | 1.21 | 1.34 |
|  | 1.00 | 1.22 | 1.14 | 1.1 |
| *SREBP-1C* | 1.00 | 2.66 | 2.23 | 1.98 |
|  | 1.00 | 3.01 | 2.75 | 2.29 |
|  | 1.00 | 2.95 | 2.13 | 1.77 |
| *FAS* | 1.00 | 1.33 | 1.12 | 1.02 |
|  | 1.00 | 1.45 | 1.08 | 0.89 |
|  | 1.00 | 1.24 | 1.24 | 1.15 |
