# Supplementary material for: Green tea polyphenol treatment attenuates atherosclerosis in high-fat diet-fed apolipoprotein E-knockout mice via alleviating dyslipidemia and up-regulating autophagy
Source: PLoS One. 2017 Aug 4;12(8):e0181666. doi: 10.1371/journal.pone.0181666 (PMC5544182; doi:10.1371/journal.pone.0181666)
Supplement: S13 Table — (DOC) [file pone.0181666.s013.doc]

**S13 Table. Effects of green tea polyphenol on protein expressions**

|  | C57BL/6J/Control group | ApoE^-/-^/Control group | ApoE^-/-^/GTP-L group | ApoE^-/-^/ GTP-H group |
| --- | --- | --- | --- | --- |
| LC3 | 1.00 | 0.74 | 0.91 | 1.21 |
|  | 1.00 | 0.65 | 1.06 | 1.15 |
|  | 1.00 | 0.76 | 1.12 | 1.25 |
| PPARα | 1.00 | 0.68 | 0.79 | 0.98 |
|  | 1.00 | 0.62 | 0.68 | 0.91 |
|  | 1.00 | 0.57 | 0.75 | 0.93 |
| p62 | 1.00 | 0.52 | 0.82 | 0.71 |
|  | 1.00 | 0.56 | 0.65 | 0.75 |
|  | 1.00 | 0.62 | 0.72 | 0.84 |
