# Supplementary material for: Green tea polyphenol treatment attenuates atherosclerosis in high-fat diet-fed apolipoprotein E-knockout mice via alleviating dyslipidemia and up-regulating autophagy
Source: PLoS One. 2017 Aug 4;12(8):e0181666. doi: 10.1371/journal.pone.0181666 (PMC5544182; doi:10.1371/journal.pone.0181666)
Supplement: S14 Table — (DOC) [file pone.0181666.s014.doc]

**S14 Table. Effects of green tea polyphenol on serum oxLDL**

| oxLDL(ng/mL) | C57BL/6J/Control group | ApoE^-/-^/Control group | ApoE^-/-^/GTP-L group | ApoE^-/-^/ GTP-H group |
| --- | --- | --- | --- | --- |
| Mean | 41.58 | 124.39 | 87.32 | 79.57 |
| SD | 5.58 | 10.16 | 11.76 | 9.08 |
