# Supplementary figures and images for: Green tea polyphenol treatment attenuates atherosclerosis in high-fat diet-fed apolipoprotein E-knockout mice via alleviating dyslipidemia and up-regulating autophagy
Source: PLoS One. 2017 Aug 4;12(8):e0181666. doi: 10.1371/journal.pone.0181666 (PMC5544182; doi:10.1371/journal.pone.0181666)

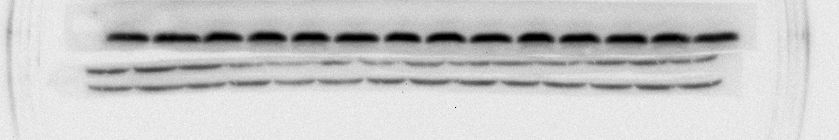

Supplement: S1 Fig — (TIF) [file pone.0181666.s015.tif]

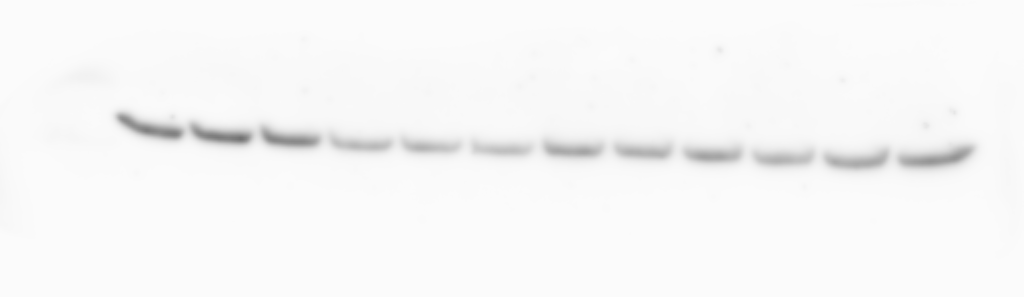

Supplement: S2 Fig — (TIF) [file pone.0181666.s016.tif]

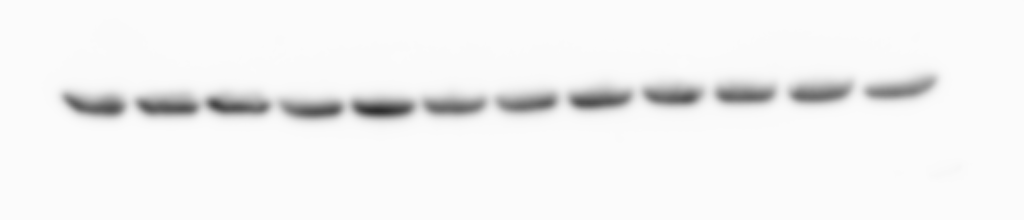

Supplement: S3 Fig — (TIF) [file pone.0181666.s017.tif]

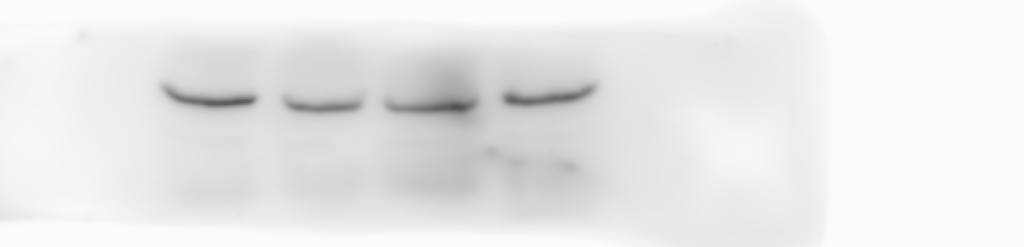

Supplement: S4 Fig — (TIF) [file pone.0181666.s018.tif]

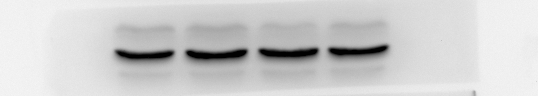

Supplement: S5 Fig — (TIF) [file pone.0181666.s019.tif]
